# Self-Assembled Metal Nanohole Arrays with Tunable Plasmonic Properties for SERS Single-Molecule Detection

**DOI:** 10.3390/nano12030380

**Published:** 2022-01-24

**Authors:** Daniela Lospinoso, Adriano Colombelli, Mauro Lomascolo, Roberto Rella, Maria Grazia Manera

**Affiliations:** CNR-IMM, Institute for Microelectronic and Microsystems, Lecce, University Campus Ecotekne, Via per Monteroni, 73100 Lecce, Italy; adriano.colombelli@le.imm.cnr.it (A.C.); mauro.lomascolo@cnr.it (M.L.); roberto.rella@cnr.it (R.R.)

**Keywords:** metal nanoholes array, nanofabrication, nanosphere lithography, near field characterization, SERS

## Abstract

Arrays of metal nano-holes have proved to be among of the most promising structures for applications in the field of nano-photonics and optoelectronics. Supporting both localized and propagating surface plasmons resonances, they are characterized by very high versatility thanks to the tunability of these modes, by means of the change of their periodicity, the size of the holes and metal composition. The interaction between different optical features can be exploited to modulate electromagnetic field distribution leading various hot-spots excitations on the metal surfaces. In this work, long range ordered arrays of nano-holes in thin gold films, with different geometrical characteristics, were fabricated by a modified nano-sphere lithography protocol, which allows precise control on holes’ dimensions together with the preservation of the order and of the pristine periodicity of the array. An in-depth analysis of the correlation between surface plasmon modes interference and its effect on electromagnetic field distribution is proposed, both by numerical simulations and experimentally. Finally, metal nano-holes arrays are exploited for surface enhanced Raman experiments, evaluating and comparing their performances by the estimation of the enhancement factor. Values close to the single molecule detection are obtained for most of the samples, proving their potentialities in surface enhanced spectroscopy applications.

## 1. Introduction

Plasmonic nanostructures with tunable optical properties have many applications in the field of nanophotonics and optoelectronics for applications in sensitive and label-free biological sensing of various molecular compounds [1,2,3,4]. Important implications can be found in medical research [5,6] but also for environmental monitoring [7] and spectroscopy [8,9]. The excitation of the surface plasmonic (SP) modes leads to a strong concentration of light energy in nanoscale volumes and, in parallel, increases the intensity of the near optical field by several orders of magnitude with respect to exciting field. In order to be detected, biorecognition events must occur within this SP-enhanced optical near field regions, predominantly localized in small geometric features (sharp tips, nanogaps). These phenomena are the main mechanism responsible for the surface enhanced spectroscopies such as Raman spectroscopy (SERS).

In conventional SERS transducers, colloidal plasmonic nanoparticles are typically employed to create strong “hot spots’’ regions. Here nano-gaps are created between the aggregate or the assemblies of the patterned surfaces. An ideal SERS substrate must guarantee high enhancement field for Raman signal enhancement together with uniform and reproducible response so that large areas with regular hotspots are needed. However, although SERS spectroscopy based on random aggregates of metal nanoparticles or nanowires has shown highly sensitive detection capabilities, the non-uniform distribution of “hot spots” and the consequent difficulty in the positioning of probe molecules at their sites, does not allow for a controlled and reproducible response. Moreover, an easy integration with microfluidics tools and other biological assay results very difficult in that context, together with uniform reproducible responses over a large area.

Design strategies for the realization of substrate-supported nanomaterials and nanostructures to plasmonically enhance optical sensing signals is then highly required. A controllable nanometer-scale engineering of geometrical and compositional properties of nanomaterials is essential to get a fine tuning of plasmonic resonances and electromagnetic field localization at the subwavelength scale. This can be the key for the realization of optimized and tuned optical transducers for all surface enhanced spectroscopies [10].

The interference between different plasmonic modes on the same surface can be a possible route: intercoupling of localized SPs among aligned metal NPs of decreasing distance is one possibility [11]. Interference and eventually coupling between SP modes of different nature (such as propagating and localized SPs) is an open field worth of investigated holding great promise for SERS applications.

An easy realizable route is represented by ordered arrays of nanometric holes in metallic thin films; very promising structures for applications in light manipulation [12]. Supporting localized surface plasmons (LSPs), propagating surface plasmons (also referred to as surface plasmon polaritons SPPs) resonances as well as diffractive optical modes due to their periodic arrangement, metal nano-hole arrays are the most versatile plasmonic nanostructures. It is possible to modulate the intensity and the spectral position of these modes across the visible to near-infrared range by changing the periodicity, size of the hole, and metal composition, making them the proper choice both for refractive index sensing and surface-enhanced spectroscopies applications [13,14,15]. The interaction between the involved modes can also be properly engineered for a fine-tuning of electromagnetic field distribution and confinement on the metal surface or inside the holes.

Different authors have observed changes in far field optical properties to due structural variation in NHA [13,16,17,18], sometimes getting contrasting results: blue or red shift at the increasing or decreasing of the hole diameter without a deep analysis of the reasons of achieved different outcomes.

In addition, due to their superior scattering cross-section with respect to corresponding nanodisks of the same size, metal nanoholes arrays have been proposed in the literature as SERS substrates. Silver is preferred as most efficient metal in visible to this purpose in different combinations of diameters and hole spacing. The reported Raman enhancement factors are around 10^4^–10^6^ [18,19,20,21], being the upper limit often obtained after introducing some roughness on the metal surfaces to increase scattering with evident problems of reproducibility.

Some attempts to exploit gold as a chemically stable material have been proposed without noticeable improvements in enhancement factors values (EF = 4 × 10^5^) [22]. Further examples [13,23,24,25] in this context have greatly contributed to understanding of the role of near and far field properties in the achievement of Raman signals, but with less analytical information when compared with the literature.

A different strategy [26] was the coupling of localized plasmonic modes of Au nanostars with those excited on an ordered array of nanoholes in thin Au films via DNA hybridization. Besides the large enhancement of the electromagnetic field because of the strong coupling between the involved plasmonic modes, an enhancement factor of 4.5 × 10^6^ can be just achieved despite the laborious route achieved.

An alternative interesting issue is the investigation of the optical properties of a bare Au nanohole array trying to push its analytical performance at its limit while playing with just a few geometrical parameters: thickness and hole diameters.

This work intends to fill this gap offering a deep insight into the different modes they can support to find the proper combination of optical and plasmonic modes and their eventual interference resulting in a fine distribution and confinement of the enhanced electromagnetic fields over the nanometal surfaces in the near field and reliable correlation with far field measurements. The ultimate aim is the realization of SERS transducers where uniform distribution of hotspots on the active nanoarray areas can create ideal conditions for high and reliable Raman enhancement factors avoiding the employment of laborious sandwich strategies. Relative spectral positions of the involved hybrid modes with respect to excitation and Raman band is considered as well, such that both the incident and Raman-scattered fields are effectively enhanced.

To this purpose an easy and inexpensive nanofabrication technique, able to realize a long-range ordered array of nanostructures with accuracy and flexibility at the same time is required.

Conventional photolithography is unable to get the required nanoscale features. Some of the suitable techniques to overcome its limitations would be electron-beam lithography [27], dip-pen lithography [28], and focused-ion lithography [29]. However, these are not always available, as well as being expensive and time-consuming. Alternative techniques include anodic aluminum oxide (AAO) template-based lithography [30], laser interference lithography [31] and nanosphere lithography (NSL) [32]. The latter has become a suitable large area nanofabrication technique due to its inexpensiveness, high resolution, and flexibility to tune nanostructure size and shape. The NSL technique consists of the arrangement spherical colloids onto a substrate to form a close packed monolayer [33]. This monolayer can be used as it is as a sacrificial mask to deposit metal and obtain a basic nanostructure. Furthermore, different processing steps, such as plasma etching or thermal treatment, allow us to modify it to obtain an array of nanostructures of different shapes. Used as it is, the mask allows obtainment of nano-prisms arrays, while, by reducing the diameter of the spheres, nano-hole based arrays can be fabricated by depositing a metal layer by conventional deposition techniques (thermal evaporation, electron beam or sputtering) through the interstitial spaces of the mask, and then by removing the mask [13,34]. A useful guide for this technique that explors the unique properties of exotic metamaterials for different technological applications in the field of plasmonics, SERS, and energy harvesting can be found in Liang et al., 2019 [35].

As a step forward with respect to our previous work [34], in this paper, a very efficient and reproducible fabrication protocol based on a modified NSL method is presented. Highly ordered nano-holes arrays of different size and diameter in thin gold films with tuning plasmonic features have been realized on centimeter-sized area of metallic film. By combining dry etching processes of self-assembled monolayers of polystyrene colloids with metal physical deposition, interesting NHA design can be explored, including thickness, hole shape, diameter, and lateral periodic or quasi-periodic spacing.

The relationship between the geometry of the different types of nanostructures and optical phenomena associated with them, such as enhanced absorption or extraordinary transmission, are analyzed in detail. The theoretical and experimental investigation and comparison of the optical characteristics of four types of structures, with two different diameters and thickness, highlight their hybrid nature due to the interaction between diffractive and plasmonic modes whether localized and propagating. Amplifications and local confinement of the electric fields are found, due to the interaction with light, and show potential to modulate their optical properties according to their geometric characteristics. The nanostructures were optically characterized in view of their employment as optical transducers in nanoplasmonic chemosensors platforms working in the UV-vis spectral range. Their functional abilities such as surface enhanced Raman spectroscopy (SERS) substrates are explored and compared, both theoretically and experimentally, to find the best configuration to achieve single-molecule detection.

The drown route can be useful to orient the design of further functional nanostructures of interest for other surface spectroscopies applications.

## 2. Materials and Methods

### 2.1. Nano-Holes Fabrication

The first step in ordered metal nano-holes fabrication consists in the self-assembly of polystyrene (PS) nanospheres at air/water interface for the realization of a close-packed array (CPA) with hexagonal symmetry.

**Figure 1:**
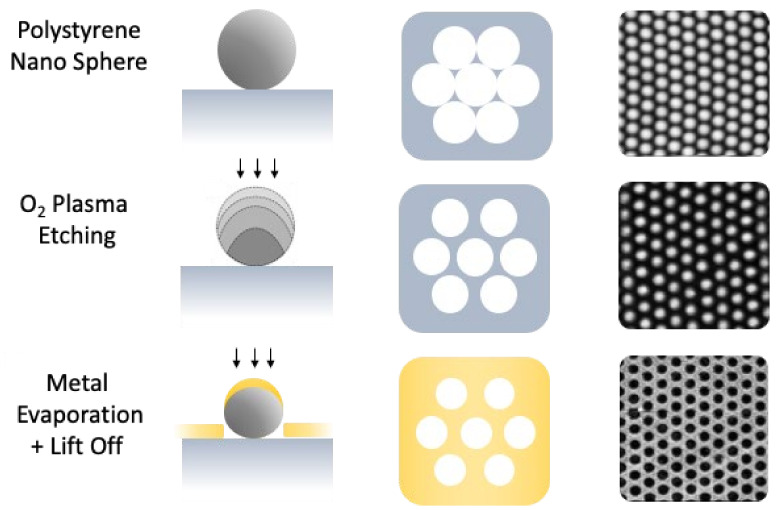
Schematic representation of the nano-hole array fabrication process: after obtaining the CPA of NPs, the size of the NPs are reduced by an oxygen plasma treatment. The NCPA thus obtained is used as a mask through which the metal is evaporated to obtain the ordered nano-holes array.

The detailed description of the technique and the home-made apparatus are reported in previous works [34,36]. Briefly, a PS particle suspension was slowly dispensed by a motorized syringe pump onto a tilted glass slide partially immersed in a water-filled Petri dish. Polystyrene particles with a nominal diameter of 500 nm were used in the experiments (Sigma-Aldrich, Darmstadt, Germany, aqueous suspensions with a concentration of 10 wt %). In order to trap the polystyrene nanosphere at the air/water interface, a mixture of alcohol and polystyrene water solution was employed. In this work, the polystyrene particle solution was diluted to a final concentration of 2.5 wt % in a 1:1 ratio with ethanol, which acted as a spreading agent on the water surface. After the close-packed array formation at the air/water interface, the colloidal mask can be deposited onto a solid substrate. A glass substrate (commercial slide) was employed, removing the water in two distinct phases, firstly by using a peristaltic pump, and secondly by self-vaporization. The next step consists in obtaining non-close packed arrays (NCPAs) of PS nanospheres. As schematically described in Figure 1, they are obtained by a post-treatment process based on oxygen plasma etching, which induces a progressive and controllable reduction of the spheres diameter, leaving the order and periodicity unchanged.

A commercially available plasma cleaning setup (Diener ATTO, Diener electronics, Ebhausen, Germany) was used to perform the treatment. Polystyrene spheres were etched using 40 W radio-frequency (RF) power oxygen plasma at a pressure of 0.25 mbar. Successive steps, with exposure times of 120 s each, interspersed with cooling pauses of 60 s, were repeated until the desired size is reached: in particular, in this work, we consider two distinct dimensions obtained with total etching times of 12 and 14 min, respectively.

The so obtained NCPAs of nano-spheres were used as lithographic masks to fabricate a planar distribution of highly ordered gold nano-holes, by adopting a two-step metal evaporation process (electron beam evaporation, EBE) (Figure 1). After a deposition of 2 nm thick titanium adhesion layer, a gold layer with a nominal thickness of 30 or 100 nm was deposited, obtaining four types of structures, in total. After the metal deposition, the colloidal mask was removed with a chemical or mechanical lift-off process. In particular, larger PS spheres were mechanically removed by a suitable adhesive tape, instead, PS spheres smaller than 200 nm were dissolved by an overnight ultrasonication bath in toluene.

### 2.2. Morphological Characterization

The morphology of the Au NHs realized on glass substrates was investigated by atomic force microscopy (AFM NT-MDT Spectralight, Moscow, Russia).

**Figure 2:**
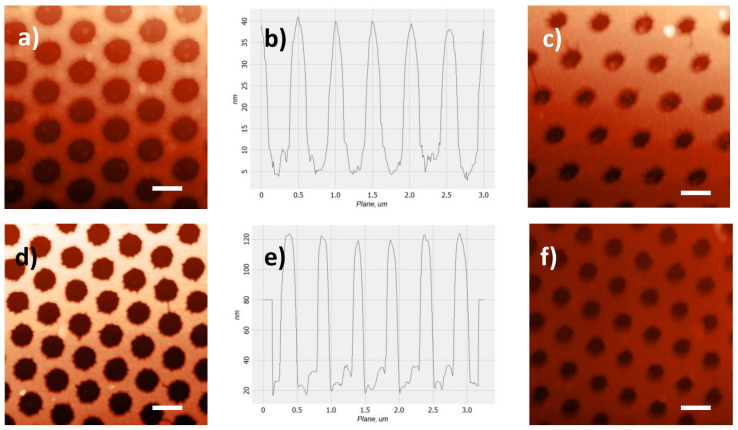
Atomic force microscopy height topographic images of the four types of samples with diameters of (**a**) (350 ± 20) nm, (**c**) (240 ± 20) nm, (**d**) (320 ± 20) nm, and (**f**) (230 ± 20) nm. (**b**,**e**) The topographic image profiles that show the height of the gold film: 30 nm for the samples in (**a**,**c**) images, 100 nm in (**d**,**f**) images. Scale bars: 500 nm.

Height topographic images (Figure 2) were acquired in semi contact mode, using commercial probes (NT-MDT AFM probes ETALON series, HA-NC: frequency 235−140 kHz, force constant 12−3.5 N/m).

### 2.3. Optical Characterization

**Figure 3:**
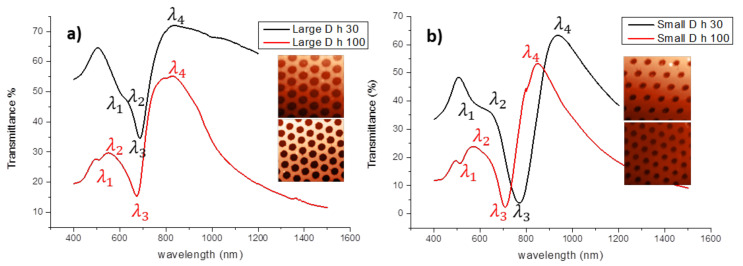
Optical transmittance spectra of the four samples in air, normalized to the signal of a bare glass substrate: (**a**) transmittance spectra of the large diameters nanoholes in gold film, 30 nm and 100 nm thick, respectively, (**b**) transmittance spectra of the small diameters nanoholes in gold film, 30 nm and 100 nm thick, respectively. λ_1_, λ_3_, indicate the weak and the very sharp minima, respectively, λ_2_, λ_4_ indicate the weak and the marked maxima, respectively.

The optical transmittance of all samples on glass (Figure 3) were measured in air at normal incidence by a Cary 500 UV-vis-NIR Spectrometer (Varian, Palo Alto, CA, USA), in the 400–1000 nm spectral range and normalized to the corresponding signal of a bare glass substrate, with a spectral resolution of 1 nm in the 400–1200 nm spectral range.

### 2.4. SERS Experiments

The experimental setup consists of an NT-MDT AFM NTEGRA SPECTRA (Zelenograd, Russia): an AFM integrated with a MicroRaman Spectra-C spectrometer (MS 3504i # 015022, SOLAR TII) and a 1024 pixels × 400 pixels CCD (Andor detector) with top laser illumination (the optical path is perpendicular to the sample). The signal collection is performed in reflection mode (backscattered signal collection mode with upright configuration).

A He–Ne source provides a linearly polarized 632.8 nm laser light as excitation source (30 mW) focused by Mitutoyo M Plan Apo 100× microscope objective (long working distance, numerical aperture 0.7) located above the sample, at ambient pressure, temperature, and humidity.

Scattered and reflected light are collected by the same objective, spectrally filtered through an edge filter (633 nm plus Rayleigh scattering), and then sent to a dispersive spectrometer (600/600 lines/mm grating).

The position of the laser spot can be precisely controlled by a mirror with a piezomotor.

Before carrying out the SERS experiments, all substrates were functionalized by immersion in a 10 mM solution of benzenethiol (BT, 97% from Sigma-Aldrich) in ethanol for a time of 15 h: sulfur–Au chemical bonds ensure the formation of a monolayer on gold nanostructures [37].

Then they were rinsed with ethanol to remove excess thiol. Pure liquid BT was measured in a glass cuvette. The selected Raman probe shows, under 633 nm excitation, the strongest anti-Stokes Raman bands between about 676 and 703 nm.

Spectral acquisitions in SERS conditions were performed on ten different points of the BT-functionalized samples, with an incident laser power of about 0.2 mW (measured with a power-meter), integration time 10 s, pinhole aperture 50 µm (corresponding to a monochromator slits size of L≅18 μm).

The spectral acquisition of BT Raman spectra in liquid was performed under the same conditions, except for the integration time that was 600 s.

### 2.5. Numerical Procedures

**Figure 4:**
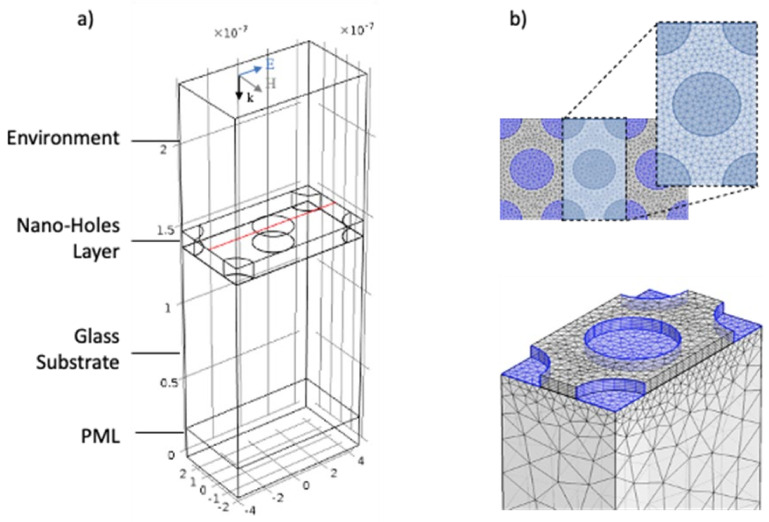
(**a**) Sketch of the geometry of the computational domain adopted in the numerical simulations; from the bottom: a perfect matched layer (PML), glass substrate, gold nano-holes and environment. (**b**) Representation of the unit which simulates the fabricated nano-structures: a free triangular mesh, utilized for the glass substrate and the environment, and a swept mesh, chosen for the nano-structure and the PML, are visible.

A COMSOL Multiphysics radio frequency (RF) module was used to calculate far field spectra and electric field distribution of periodic nanoholes array. It is possible to develop a 3D simulation with appropriate boundary conditions to simulate the unit cell of the analyzed system, with hexagonal symmetry of the periodic array and perpendicular excitation by linearly polarized light. The geometry of the computational domain and the nano-holes are characterized by specific dimensions derived by considering a close packed array of hexagonal distributed nano-spheres with diameter of 500 nm. Starting from the bottom of the computational domain reported in Figure 4a, the first domain is a perfectly matched layer (PML) followed by the glass substrate, on which the gold nano-holes have been modeled. As reported in Figure 4b a cylinder characterized by a perfectly circular shape and a thickness of 30 or 100 nm, to simulate the fabricated nano-structures, was chosen. The last domain at the top represents the external environment. In the model, a free triangular mesh was used for all computational domains except for the PML and the nano-holes layer. A swept mesh was adopted for these domains, with a local refinement near the holes. The optical properties of the involved materials were described by their frequency-dependent dielectric functions. In the visible spectral range, optical properties of the nanostructured gold can be modeled by complex dielectric constant value, whose real and imaginary components can be described by an interpolated version of the experimental data from Johnson and Christy. Surface plasmon activation was achieved using the wavelength modulation technique, simulating a linearly polarized electromagnetic wave coming from the top of the geometry which represents the external environment. Boundary conditions were set for both the upper and lower edges of the simulation domain, in order to calculate the reflection and transmission coefficients of the system. Perfect electrical conductor (PEC) and perfect magnetic conductor (PMC) boundary conditions were used on the sides of the unit cell to simulate an infinite array of plasmonic nanostructures with hexagonal geometry. Far and near field analysis were indeed performed simulating all the four samples, setting the corresponding thicknesses and diameters.

## 3. Results and Discussion

### 3.1. Optical and Morphological Correlation

By reducing the diameter of the spheres and by depositing 30 or 100 nm of gold, respectively, we were able to obtain the four samples investigated in this work.

Their AFM images, after the lift-off process, are reported in Figure 2. Figure 2a,d shows samples with a diameter of $\left(350\;\pm \;20\right)\;\mathrm{nm}$ and $\left(320\;\pm \;20\right)\;\mathrm{nm}$ (hereinafter referred to as large D), respectively; Figure 2c,f shows samples with a diameter of $\left(240\;\pm \;20\right)\;\mathrm{nm}$ and $\left(230\;\pm \;20\right)\;\mathrm{nm}$ (hereinafter referred to as small D), respectively. Figure 2b,e represents topographic image profiles that show the height of the gold film: 30 nm for samples represented in the top row images, 100 nm in the bottom one.

From the AFM images it can be seen that the high order of the structures is preserved even in samples with smaller diameters, whose masks have therefore undergone a more thorough reduction treatment.

Transmission spectra (Figure 3) show very interesting characteristics that can be related to the coupling of light to the periodic structure and to the nanometric dimensions of the holes.

It should be emphasized that the observed minima and maxima, in the far field spectra, are the results of the excitation of hybrid modes resulting from the contribution of different optical and plasmonic modes and of their eventual interference. Prevalence of one over the others is dictated by the periodical and geometrical features of the array.

The idea to couple LSP and SPP modes by exploiting periodic arrangements of metal nanostructures close to thin metal films has already been reported [38,39]. As expected [40,41], the resulting highly regular hotspots patterns, uniformly distributed in the near field, as verified by numerical tools, contribute to a great enhancements in Raman signals even if just theoretically demonstrated.

Therefore, here, the coexistence of different modes and their tunability is seen as a useful method to properly arrange hotspots on the holey metal surfaces to maximize their analytical performance as SERS substrates. A brief description of the involved modes are presented below trying to draw their contribution to far and accordingly near field.

The periodicity allows the formation of the so-called surface plasmon polaritons (SPPs): the grating-like nature of the nanoholes arrays provide the extra momentum needed for free space coupling. One can refer to them as SPP-Bloch waves (SPP-BW), i.e., standing waves consistent with Bloch’s theorem [42], that give rise to one or more pronounced peaks that can be related to the extraordinary optical transmission (EOT) through subwavelength apertures in metal films which occurs both for hole arrays and single apertures surrounded by periodic corrugations [43]. This phenomenon occurs when the SPP-BW modes on the two sides of a holes array interact, re-radiating the incident field and increasing the optical transmission [44,45].

For hexagonal arrays of nanoholes, the resonance condition can be written as [46].

(1) $$\lambda_{SPP}\left(i,j\right)=\frac{\sqrt{3}}{2}\frac{P}{\sqrt{i^{2}+ij+j^{2}}}\sqrt{\frac{\varepsilon_{m}\varepsilon_{d}}{\varepsilon_{m}+\varepsilon_{d}}}\;$$

where $\lambda_{SPP}$ represent the SPP resonant wavelength, P is the lattice spacing of the nanohole array, i and j are integer indexes of the peaks which represent the Bragg resonance orders of the array, $\varepsilon_{m}$ and $\varepsilon_{d}$ are the real parts of the relative permittivity of the film material (in our case gold) and the surrounding environment (the glass substrate and air in our experiments).

In ultrathin (<100 nm) symmetrically bound (i.e., surrounded by the same dielectric medium) metal films, the two SPPs combine to form two coupled SPPs, the long range surface plasmon polariton (LR SPP) and the short range surface plasmon polariton (SR SPP), which correspond to the higher wavelength mode [47], whose location can be predicted by:

(2) $$\lambda_{\;\pm \;}≅\frac{\lambda_{SP}}{\sqrt{1\;\pm \;e^{-k_{SP}d}}}$$

According to this equation, together with the previous one, it was possible to establish that the strongest transmission peak $\lambda_{4}$, that appears in the spectra in Figure 3, could be assigned to the SR SPP (1,0) while the weaker peak $\lambda_{2}$ to the LR SPP (1,0).

The marked difference in intensity between the two peaks can be attributed to the asymmetry of the metal surrounding environment which causes the suppression of the LR SPP [48].

It is worth to notice that, as already observed elsewhere [49], increased absorption rather than transmission (EOT) is observed in case of ultrathin film nanohole arrays.

It can be also noticed that peaks become more pronounced and shifted to lower wavelength as the thickness of the gold film increases, because of the uncoupling of the SPP modes at the two metal interfaces.

Ordered nanoholes also support other modes due to the so-called Rayleigh–Wood’s anomalies (RWA) that give life to rather weak figures compared to plasmon modes, visible as minima in the transmission spectra, but sometimes covered by other processes.

The relation for the free-space incident wavelength of a RWA, in the case of hexagonal arrays, is:

(3) $$\lambda_{min}\left(i,j\right)=\frac{\sqrt{3}}{2}\frac{P}{\sqrt{i^{2}+ij+j^{2}}}\sqrt{\varepsilon_{d}}$$

According to this equation, the $\left(1,0\right)$ RWA minimum at glass interface should be at 650 nm, and may contribute to the hybrid $\lambda_{3}$ mode in the 350 nm diameter samples (Figure 3a). It is a diffractive mode that depends only on the periodicity and relative permittivity of the surrounding medium, therefore it does not undergo any displacement as the metal thickness varies, as can be observed in Figure 3a.

Moreover, even if in the arrays of metal nanoholes the mechanism that governs resonances is mainly related to the SPP, a crucial role is played by excitation of dipolar localized surface plasmon (LSP) resonance at the hole circumference. The optical response of holes strongly depends on their shape and size compared to the wavelength of light, as well as on the metal thickness and on the surrounding dielectric media [50].

Localized resonances influence the position of the transmission maximum which shows a large red-shift from the cutoff condition, more evident in the thin film regime, and as the dielectric constant of the substrate increases [50].

As the resonances associated with surface plasmons localized on holes can be predicted from known LSPR frequencies of metal particles with the complementary shape [47], the minima indicated with λ_3_ in Figure 3b can be identified with the main dipole LSPR of the holes. Moreover, as it can be observed in the same figure, in thinner holey films, the position of the minimum experienced a red-shifting, due to the occurrence of interfering propagating modes likely promoted by the close distance between the two dielectric interfaces. This interpretation is confirmed also by near field simulation shown in the following Figure 4, reporting a clear difference in the distribution of the electric field over the metal surface and on the holes edges for the above mentioned samples.

The spectral locations of LSPR optically excited on arrays of nanoparticles or nanoholes are expected to also vary depending on the height of the nanostructures [51]; accordingly, the position of the minimum smaller holes samples are found to blue shift as the thickness of the film increases (Figure 3b).

The analogous mode for the larger hole samples (Figure 3a) is expected to be located in the infrared region, thus not visible in the investigated set of samples.

### 3.2. FEM Results

**Figure 5:**
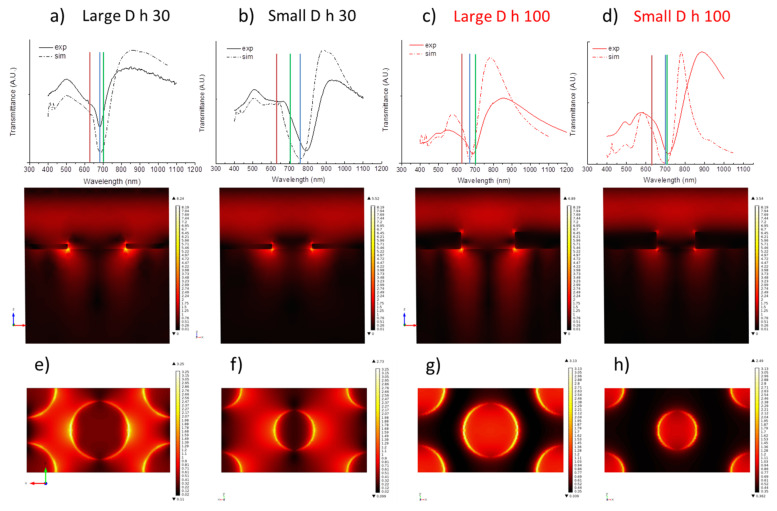
(**a**–**d**) Simulated (dashed lines) and experimental transmittance spectra showed together for comparison. Vertical lines indicate the position of the excitation wavelength (red), the strongest minimum wavelength λ_3_ (blue), and the Raman band position of the selected Raman probe (green). (**e**–**h**) Vertical (top) and horizontal (bottom) cross sections of the local fields distribution at the strong minimum position λ_3_.

The calculated transmittance spectra are shown in Figure 5a,b relating to a 30 nm thickness, and in Figure 5c,d relating to a 100 nm thickness, for large and small diameters, respectively, as a dashed line together with the experimental ones (solid line). As one can see by comparing the calculated and experimental curves, not only the overall trend is maintained, but also, all experimental resonances can be found in the calculated curves. However, the calculated resonance figures are more pronounced than the experimental counterparts. Moreover, the strongest transmission peak $\lambda_{4}$ is slightly blue-shifted for all the samples, except for the first one (large D, h 30, Figure 5a).

These discrepancies are widely expected, due to the inevitable differences between the model and the real structures, of which the main concern is the fact that the modeled structures have clear-cut profiles, whereas the real ones are smooth. Moreover, the experimental transmittance is integrated on a sample surface that contains many domains with different orientations, while the model consists of a single excited domain with linearly polarized light.

The hybrid mode represented by the minimum in transmittance λ_3_ is expected to contribute more to the performance of the nanostructured layer, due to the close spectral position to the incident light wavelength (He–Ne laser, 632.8 nm). Therefore, a more detailed investigation of the electric field near field distribution over the holey surfaces when the mode is excited is reported.

Vertical (top row) and horizontal (bottom row) cross sections of the local fields distribution at the minima position λ_3_ (Figure 5e–h, top and bottom, respectively) evidence the confinement of the field near the rim of the holes at glass and air interfaces, and along their wall. The electric field intensity on the rim of the holes is almost similar for all investigated substrates, while noticeable differences can be evidenced in the intensity distribution over the metal surface between the holes and on their vertically walls, concentrating at metal-glass interfaces.

This is evidenced particularly in thinner holey arrays, where the hybrid nature of the mode is more evident due to the stronger contribution of diffractive and propagating modes extending over the metal surfaces at the two dielectric interfaces. Thicker films, on the other hand, confine the enhanced electric field exclusively to the edges of nanoholes due to the stronger contribution of localized dipolar modes.

The two different electric field distributions are responsible of different far field optical responses reported in Figure 3 and of their functional ability in detecting molecules by spectroscopic tools.

Defining the proper configuration for exploiting the nanostructures as SERS substrates is worth of investigation to orient the nanofabrication in the correct direction.

Once defined an analytical parameter for comparing SERS abilities of the investigated nanostructures also the relative position of SP band with respect to incident light wavelength and Raman band spectral position will be considered as suggested by Van Duyne et al. [52]. To this purpose, vertical lines in transmittance spectra of Figure 5a–d are reported as a guide for the eye to evidence excitation wavelength (red), the strongest minimum wavelength λ_3_ (blue), and the Raman band position of the selected Raman probe (green).

### 3.3. SERS Performances

To evaluate and compare the SERS performance of different substrates the so-called enhancement factor (EF) is used.

Defined by the ratio $\sigma_{SERS}/\sigma_{Raman}$ between SERS and Raman differential cross sections, the most widely used formula to estimate the average SERS EF is:

(4) $$EF=\frac{S_{SERS}/N_{SERS}}{S_{Raman}/N_{Raman}}$$

where $S_{SERS}$ and $S_{Raman}$ are the integrated SERS and Raman signals of the Raman probe molecules, acquired under the same experimental conditions (or, eventually, normalized in respect of the different conditions, e.g., acquisition time), $N_{Raman}$ is the average number of molecules in the scattering volume for the Raman experiment, $N_{SERS}$ is the average number of adsorbed molecules in the scattering volume, which, in the SERS experiments, is often considered as a surface.

**Figure 6:**
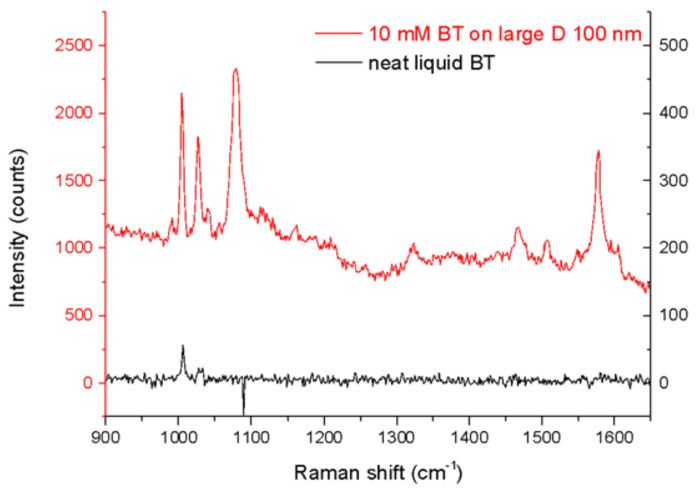
Examples of BT-functionalized Au NHs array (large diameter, 100 nm thick) (red line), and of liquid BT in glass cuvette (black line) Raman spectra.

In Figure 6, an example of spectra acquired during SERS experiments are shown: the black line represents the Raman spectrum of liquid BT, while the red one is due to the Raman signal being enhanced by one of the nanostructures (large D, h 100). It can be noticed the huge amplification of the signal thanks to the SERS mechanism, compared to the signal of pure Raman active substance which shows only one weak visible peak emerging from the background.

The definition provided in Equation (4) requires an accurate estimation of $N_{SERS}$ and $N_{Raman}$ and, therefore, an accurate estimation of the surface area of the nanostructure together with the determination of the scattering volume for the system used [53].

In fact, Equation (4) can be rewritten as:

(5) $$EF=\frac{S_{SERS}^{BT}}{S_{Raman}^{BT}\;}\frac{C_{V}^{BT}\;}{C_{S}^{BT}\;}\frac{A_{eff}}{A_{eff}}H_{eff}$$

where $C_{V}^{BT}$ is the molecular density of liquid BT and $C_{S}^{BT}$ is the surface molecular density of BT on the substrate.

The scattering volume $\;V_{eff}$ is defined as a cylinder of effective base $A_{eff}$ and effective height $H_{eff}$, which, with laser intensity and collection efficiency uniform and equal to their peak values, produces the same Raman scattering at the detector as the one produced in the reality, in which these parameters are not constant.

In general, $A_{eff}$ does not correspond to the laser beam size at the focus (called beam waist $w_{0}$). In fact, $A_{eff}$ also depends on the monochromator slits width: when the slits are narrowed and the Raman signal is partly cut, $A_{eff}$ is consequently reduced.

In our case, Raman and SERS experiments are performed under the same conditions, such as laser power, objective, slit size, so, in Equation (5), $A_{eff}$ can be simplified, obtaining:

(6) $$EF=\frac{S_{SERS}^{BT}}{S_{Raman}^{BT}\;}\frac{C_{V}^{BT}\;}{C_{S}^{BT}\;}H_{eff}\frac{1}{F}$$

The so-called filling factor F is a geometrical factor which takes into account the real morphology of the SERS substrate and represents the percentage of gold surface available for the formation of the Raman probe monolayer, in respect of the total sample surface.

For the $N_{SERS}$ estimate we use a value for the surface molecular density of BT $C_{S}^{BT}=6.80\cdot 10^{14}\;\mathrm{molecules}\cdot {\mathrm{cm}}^{-2}$ reported in the literature [53] considering a close packed arrangement on a flat substrate, while the molecular density of liquid BT was calculated by $C_{V}^{BT}=\frac{\rho N_{A}}{MW}=5.89\cdot 10^{21}\;\mathrm{molecules}\cdot {\mathrm{cm}}^{-3}$, where $\rho =1.077\;g\cdot {\mathrm{cm}}^{-3}$ is the density of liquid BT at 20 °C, $N_{A}$ is the Avogadro number, $MW=110.18\;g\cdot {\mathrm{mol}}^{-1}$ is BT molecular weight.

$H_{eff}$ was experimentally determined, obtaining a value of $H_{eff}=\left(15\;\pm \;2\right)\;\mu m$, for the setup parameters adopted in SERS and Raman experiments (in particular, laser power 0.2 mW and monochromator slits size L≅18 μm).

We calculated the EF for three out of four samples that showed a huge enhancement of Raman signal (Table 1), while the small diameter—h=30 nm sample did not show any amplification of the signal which results therefore not detectable at the concentration used.

The most enhanced Raman bands were observed to be the 1080 ${\mathrm{cm}}^{-1}$ or the 1575 ${\mathrm{cm}}^{-1}$ band (approximately corresponding to 679 and 703 nm, respectively, with an excitation of 633 nm); in fact, they place themselves between the excitation and the most pronounced plasmon resonance wavelength of the different substrates.

Therefore, for all the samples, the average integrated intensity of the 999 cm^−1^ band of BT adsorbed on the Au surface (in-plane bending, $\beta_{CCC}$) was considered for the estimation of the value $S_{SERS}^{BT}$ [54,55]. $S_{Raman}^{BT}$ was calculated as the integrated intensity of the same band of the liquid BT.

The EF values reported in the table are also corrected for reflection losses and refractive index at the excitation wavelength [53].

As one can see comparing Figure 5 with the values reported in Table 1, the best SERS performances are reached by the nanostructure which shows a resonance (blue line) located between the excitation (red line) and the Raman band (green line) [52]. The only structure that does not have this feature (small diameter, h=30 nm), does not show any enhancement: in fact in this sample the resonance is located beyond the Raman line, on the opposite side with respect to the excitation.

The other three structures show very good performances, more than two orders of magnitude higher than those of similar structures reported in literature [19], very close to single molecule detection capabilities [56]. In particular, the closer the resonance and the Raman line are, the better the performance, reaching its maximum in the small D 100 nm sample, where the two lines almost overlap.

The occurrence of hybrid modes allowed the distribution of the field density besides metal surfaces also at the edges of the nanoholes where the majority of the electromagnetic enhancement originate. This uniform distribution of hot-spots has demonstrated as a smart strategy for obtaining high and reproducible SERS enhancements, but this is not just the only the requirement to pursue.

It is worth to note that the functional abilities of the investigated nanostructures as SERS substrates are the resultant of different aspects: near field distribution over the metal surfaces hosting the Raman probe molecules as well as the relative spectral position of the incident light, of the plasmon modes responsible of the electromagnetic enhancement in the near field and of the investigated Raman band.

These findings can offer a valid help in designing and developing smart SERS substrates based on metal nanohole array nanostructures.

## 4. Conclusions

By means of a modified nanosphere lithography-based technique, we were able to obtain highly ordered arrays of metal nano-holes in thin metal film on a very large area are obtained in a fairly simple, fast and low-cost way. Optical characterizations, confirmed by numerical simulation, revealed a variety of spectral features, mainly derived from excitation of different plasmonic modes, easily tunable by changing the geometric parameters of the nanostructures. The electromagnetic field distribution modulation of four types of nano-holes arrays, obtained by different thickness of the metal film or diameter of the holes, was studied experimentally and theoretically, demonstrating how it is possible to modulate plasmonic and diffractive modes coupling just as easily. The potentiality of the realized nanostructures as highly sensitive SERS substrates is tested, obtaining higher performances (EF up to 10^7^) with respect to similar nanostructures reported in the literature, close to the single molecule detection. This finding has been achieved thanks to the possibility of exploiting the most effective resonances for the enhancement mechanism as well as their relative spectral position with respect to excitation wavelength and the investigated Raman band.

The low-cost and highly reproducible approach provides an opportunity for designing novel metallic nanostructures to manipulate light on the nanoscale which could also work successfully in other surface spectroscopic techniques or as sensing platforms, allowing to select the best substrate based on the spectral position of the resonances and the distribution of the electromagnetic fields.

## Figures and Tables

**Table 1:** Summary of SERS results (enhancement factor EF) for the investigated nanostructures.

2D Transducer	Thickness *h* = 30 nm		Thickness *h* = 100 nm	
Holes diameter	Large	Small	Large	Small
SERS EF	2.54 × 10^6^	none	1.36 × 10^6^	1.40 × 10^7^

## Data Availability

Not applicable.
